# Comparison of VEGF-A secretion from tumor cells under cellular stresses in conventional monolayer culture and microfluidic three-dimensional spheroid models

**DOI:** 10.1371/journal.pone.0240833

**Published:** 2020-11-11

**Authors:** Sreerupa Sarkar, Chien-Chung Peng, Yi-Chung Tung

**Affiliations:** 1 Department of Engineering and System Science, National Tsing Hua University, Hsinchu, Taiwan; 2 Research Center for Applied Sciences, Academia Sinica, Taipei, Taiwan; 3 Taiwan International Graduate Program (TIGP), Nano Science and Technology Program, Academia Sinica, Taipei, Taiwan; 4 College of Engineering, Chang Gung University, Taoyuan, Taiwan; Medical College of Wisconsin, UNITED STATES

## Abstract

Vascular endothelial growth factor (VEGF) is a major cytokine in tumor biology affecting tumor survival, aggressiveness and pro-angiogenetic activities. In addition, cellular stresses often result in aggressive pro-angiogenetic behavior in tumors. For *in vitro* study, conventional monolayer cell culture has been broadly exploited; however, it often provides limited information due to its different microenvironment from that *in vivo*. Recently, three-dimensional (3D) cell spheroid culture provides *in vivo*-like microenvironments to study tumor biology and their survival mechanisms with better predictive power. In this work, vascular endothelial growth factor of type A (VEGF-A) secretion from osteosarcoma (MG-63) cells cultured using monolayer and 3D spheroid models under two stress conditions: nutrient deficiency (reduced serum culture) and hypoxia-inducible factor (HIF) inhibition (HIF inhibitor, YC-1) are characterized and systematically compared. In order to obtain ample sample size for consistent characterization of cellular responses from cancer spheroids under the stresses and compare the responses to those from the conventional monolayer model, a microfluidic spheroid formation and culture device is utilized in the experiments. In the analysis, cell viability is estimated from captured images, and quantification of VEGF-A secreted from the cells is achieved using enzyme-linked immunosorbent assay (ELISA). The experimental results show that the viabilities decrease when the cells face higher stress levels in both monolayer and 3D spheroid culture models; however, the VEGF-A secretion profiles between the cell culture models are different. The VEGF-A secretion decreases when the cells face higher stress conditions in the monolayer cell culture. In contrast, for the 3D spheroid culture, the VEGF-A concentration decreases for low stress levels but increases while the stress level is high. The VEGF-A regulation in the 3D models mimics *in vivo* cases of tumor survival and can provide insightful information to investigate tumor angiogenesis *in vitro*. The approach developed in this paper provides an efficient method to quantitatively and statistically study tumor growth kinetics and stress responses from highly uniform samples and it can also be applied to compare the underlying biomolecular mechanisms in monolayer and 3D spheroid culture models to elucidate the effects of microenvironments on cellular response in cancer research.

## Introduction

Vascular endothelial growth factor (VEGF) is an essential signal protein produced by mammalian cells augmenting formation of blood vessels [1]. It has also been found that VEGF is a major regulatory cytokine that promotes angiogenesis in solid tumors [2–4]. Internal microenvironments of solid tumors are typically hypoxic and regulated by a special group of proteins called hypoxia inducible factors (HIFs). HIF regulates several pro-survival pathways and further affects VEGF secretion from the tumor cells. VEGF activates pro-survival signaling pathways leading to tumor growth and ultimately malignancy [5–10]. Therefore, a number of studies have been performed to investigate VEGF expression from solid tumors; it is critical and necessary to understand the intricate balance between pro-survival and anti-survival tendencies of tumors and detect potential malignancy [4, 9, 11–17].

In physiological microenvironments, tumor cells are exposed to several unfavorable or extreme conditions, generally referred to cellular stresses [18]. These stresses induce cellular responses through regulation of cytokines like VEGF often leading to malignancy [19]. Therefore, it is desired to study VEGF secretion from tumor cells under the cellular stresses to investigate their effects on cellular responses. Various cellular stresses including heat/cold shock, mitochondrial stress, oxidative stress, chemical stresses and nutrient deficiency have been intensively studied in tumor biology [18, 20–24]. Among them, nutrient deficiency has been shown to be a common stress factor affecting apoptotic signaling pathways in various tumor cells [25–28]. In addition, several critical signaling pathways of tumor cells depend on HIFs; therefore, HIF inhibition can also act as a cellular stress for tumor cells. Furthermore, HIF inhibition can also be categorized as an indirect oxidative stress due to its direct relation to tumor hypoxia [29].

In order to systematically study the tumor cell responses under different physiological and pathological conditions including cellular stresses, three-dimensional (3D) cell culture approaches have been proposed as promising *in vitro* models [30]. Among various 3D cell culture models, cell spheroid culture is physiologically and structurally more analogous to *in vivo* microenvironments of tumor than conventional monolayer cell culture. Previous studies have found that several molecular pathways work distinctively different in 3D models due to the multicellularity, structural integrity, cell-cell interactions and development of intrinsic microenvironments, which may be able to closely resemble those of solid tumors [31–33]. 3D spheroid models have been used to study cellular oxygenation [34], transcription factors like HIF-1 [35], anti-carcinogenic molecules [36–38], growth factors [39], molecular signaling [38, 40, 41] and cytokines [42, 43] of tumor cells. Consequently, characterization of VEGF expression using the spheroid models can be exploited for analysis and prediction of tumor growth and behaviors. The results can potentially improve existing clinical therapeutic strategies for cancer patients.

A number of *in vivo* studies have been conducted to explore the role of VEGF in tumor formation and progression; however, *in vitro* studies investigating VEGF expressions under cellular stresses are relatively less explored. Furthermore, systematic comparison of the VEGF-A secretion from conventional monolayer cell culture and 3D spheroid model has not been performed due to technical limitations. Cellular stresses in 3D spheroids have been observed to potentially trigger several pro survival pathways [2, 20, 25, 37, 44], where VEGF plays a major role [45, 46]. Common challenges faced in the *in vitro* 3D culture models include tedious sample handling, maintaining uniformity and stability to avoid structural disintegration of spheroids. Conventional spheroid culture methods (e.g. hanging drop and nonadherent round-bottom culture wells [47]) have limitations of low reproducibility, huge changes in cellular microenvironments, and variations between samples due to handling errors [32]. Other commercially available 3D cell culture products such as, EZSPHERE culture dishes (Asahi Glass Corporation, Japan) or Nunclon Sphera (Thermo Scientific Inc.) also face similar limitations [48]. The methods fail to mimic the physiological conditions found in naturally growing tumors, especially, the perfused microenvironment. In contrast, microfluidic device-based 3D cell culture systems provide relatively consistent and stable platforms with lower disturbances from external sources for systematic study of tumor behavior and progression under perfusion flow, more suitable for spheroid culture [49]. The advantages of controlled fluidic motions and perfusion in microfluidic devices provide spatially confined culture conditions with better scale-up capability and versatility for spheroid culture than other 3D cell culture products.

Currently, studies of VEGF secretion from the spheroids using microfluidic systems are limited to qualitative or semi-quantitative analysis based on analysis of RNA [7, 16, 17, 43] rather than direct measurement of the protein itself. Several studies using anti-cancer agents on spheroid systems have concluded that physical properties of spheroids are related to drug efficacy [50, 51]. Furthermore, recent studies have also shown that cytokine secretion profiles are different between conventional monolayer cultures and 3D culture systems [46, 52]. A tactful approach capable of direct quantitative characterization of VEGF protein is highly desired to investigate responses of multicellular spheroids under specific cellular stress conditions without delving into the intercellular variations which can be further compared to the clinical observations.

In this study, the variations of VEGF secretion between monolayer and 3D spheroid cell cultures are investigated and compared systematically under normal and stress conditions. A microfluidic device is exploited to form and culture spheroids in this work. The device provides a high-throughput, fed-batch and perfusion culture system with controlled nutrition, aeration, growth and treatment conditions for statistically significant sample size [49, 53, 54]. In the experiments, vascular endothelial growth factor of type A (VEGF-A) secretion profiles from osteosarcoma cells (MG-63) in monolayer and spheroid cultures are characterized. The MG-63 cell line is chosen as the *in vitro* model due to its ability to form compact spheroids within relatively short periods and reported HIF and cytokine activities for comparison [9, 55–58]. The consistent and reliable 3D spheroid formation and culture is performed taking advantage of the perfusion flow controlled microfluidic devices, and the cellular responses are quantified using immunoassays and image analysis.

For demonstration, the cells are cultured under normal growth conditions and two cellular stresses: nutrient deficiency and HIF inhibition in the experiments. Both conventional monolayer cell culture and 3D spheroid culture are performed to systematically compare the cellular responses in different culture formats under same culture conditions. The quantitative cell viability and VEGF-A secretion are characterized using imaging analysis and immunoassays, respectively. The results confirm the functions of the microfluidic device for 3D spheroid formation, culture and the following assays. In addition, the quantitative cell analysis results demonstrate the distinct responses from the cells culture as monolayers and 3D spheroids emphasizing the importance of 3D *in vitro* cell culture models for various biomedical researches.

## Materials and methods

### Fabrication of spheroid culture microfluidic chip

**Fig 1:**
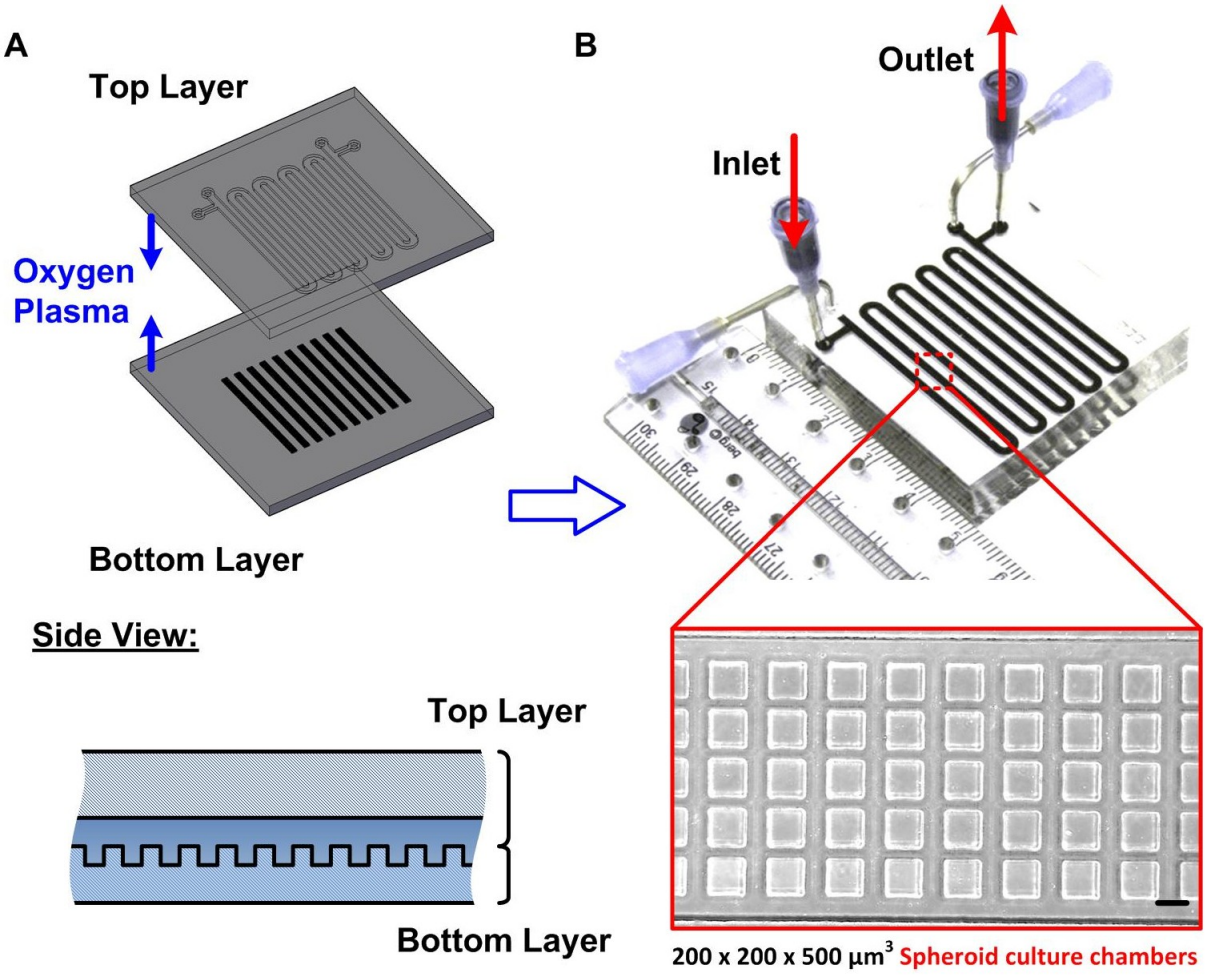
(A) Design and fabrication of the microfluidic device used for 3D cell spheroid culture. The two-layered device is made of gas permeable PDMS. The top layer is designed for fluid flow and the bottom channel is exploited for cell entrapment and subsequent formation of 3D cell spheroids. (B) Experimental photos of the fabricated device. The inset shows a brightfield microscopic image of the cell culture chambers in the bottom layer. Scale bar is 200 μm.

In this study, microfluidic devices made of polydimethylsiloxane (PDMS) are employed for 3D cell spheroid formation and culture as shown in Fig 1A [59]. This device is capable of consistently culturing 5000 spheroids, with uniformity in size without sophisticated instrumentation and tedious sample transportation [60, 61]. Due to the advantages of the device, the 3D culture model is established using the device in the experiments. The device is constructed by two PDMS layers: the bottom layer consists of 5000 cell culture chambers with approximately 200 μm and 500 μm in width and depth, respectively; and the top layer consists of a serpentine channel with 200 μm in depth covering the cell culture chambers. Two sets of inlets and outlets are designed at the ends of the serpentine channel. They are connected to blunt needles that serve as reservoirs through which cell suspensions, growth medium and other reagents are transported in and out of the device (Fig 1B). The design of the device allows for estimation of cell seeding densities, volume of medium and reagents in each cell culture chamber. In the device, each culture chamber holds approximately 0.02 μl of cell suspension solution. Also, the initial size of spheroids, can be controlled by fixing the cell seeding concentration and seeding volume that can be easily adjusted to generate optimum spheroid sizes suitable for 3D imaging along with consistent calculation of cytokine secretions during the experiments.

The microfluidic device is fabricated using a well-established soft lithography replica molding process [62]. The master molds are made using negative photoresist SU-8 2050 (Micro Chem Co., Newton, MA) on silicon wafers by conventional photolithography, followed by silanization in a desiccator to prevent undesired bonding [59]. PDMS prepolymer (Sylgard 184, Dow Corning Co., Midland, MI) and curing agent in 10:1 (w:w) ratio is then poured on top of the molds and cured in a 60°C oven overnight. Inlet and outlet holes are punched into the top channel with a diameter of 1.5 mm, and both layers are irreversibly bonded with each other using oxygen plasma surface treatment at 110 W for 40 s. The bonded device is then placed in a 60°C oven overnight to further promote the bonding and cell compatibility to complete the device fabrication process.

### Cell culture

**Fig 2:**
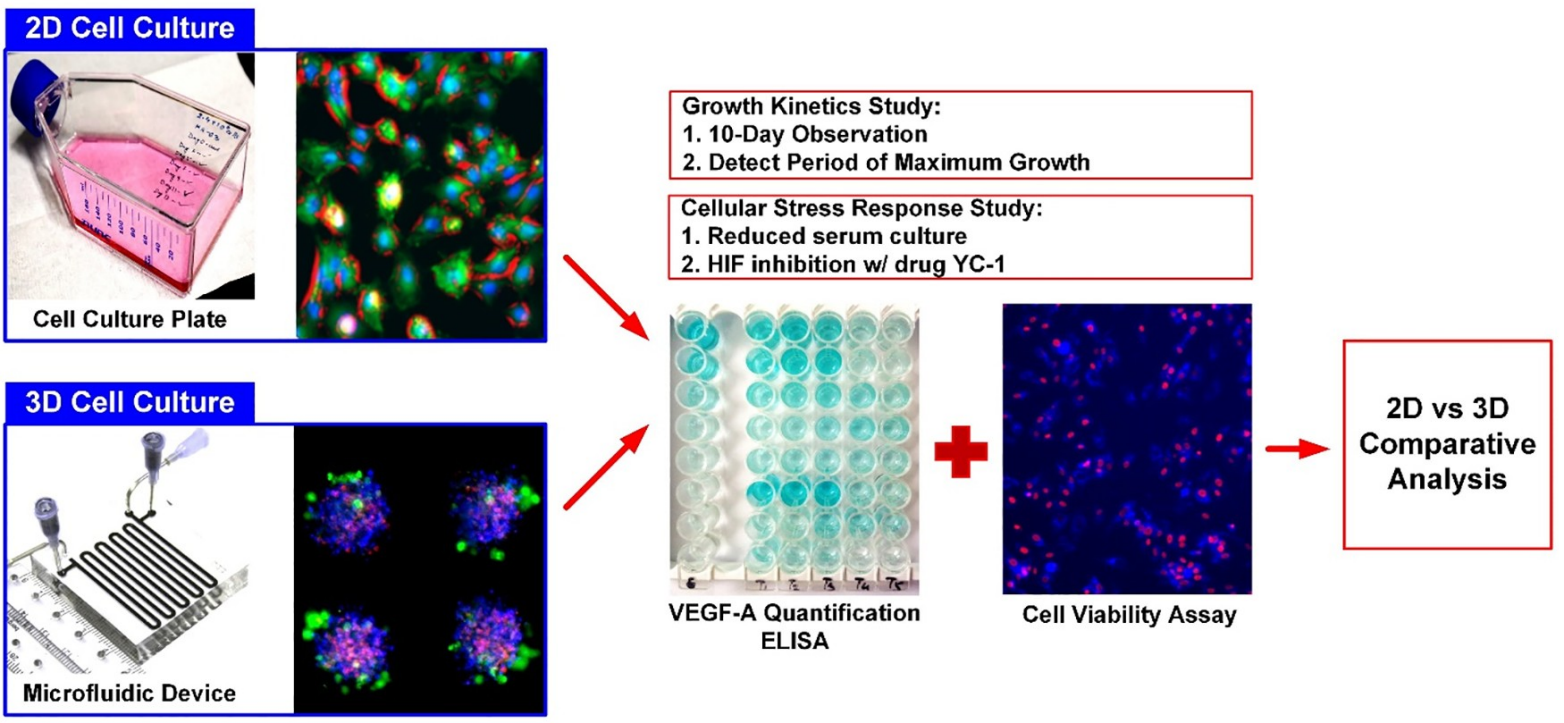
Schematics of experimental workflow. The MG-63 cells are cultured in T75 flasks until confluency, to form monolayer cell culture model while the same population of cells is used to form 3D spheroids using the microfluidic device. Conditioned mediums from the cultures are collected to quantitatively measure the VEGF-A concentration using ELISA and the cell viabilities are characterized using image analysis. The analysis results obtained from the monolayer cell culture and 3D spheroid culture models are further systematically compared.

Human osteosarcoma cells (MG-63) obtained from Bioresource Collection and Research Center (BCRC) (Hsinchu, Taiwan) are used as the model cell line in the experiments. The growth medium is composed of Minimum Essential Medium (MEM) (Gibco 41090, Invitrogen Co., Carlsbad, CA) with 10% (v/v) fetal bovine serum (FBS) (Heat Inactivated FBS, Gibco 10082, Invitrogen), 1% (v/v) antibiotic-antimitotic (Gibco 15240, Invitrogen), 1% (v/v) sodium pyruvate (Gibco 11360, Invitrogen) and 1% (v/v) non-essential amino acids (Gibco 11140, Invitrogen). The stocks are maintained in T-75 cell culture flasks (Nunc 156367, Thermo Scientific Inc., USA) in a 37°C humidified incubator with 5% CO_2_, TrypLE^™^ Express Enzyme (1X) (Gibco 12604, Invitrogen) is used to dissociate and subculture the cells. In the experiments, the 3D cell spheroid culture and conventional monolayer cell culture models are established to study their responses under different cellular stresses as shown in Fig 2.

#### 3D cell spheroid culture

In order to establish the 3D cell spheroid culture model, a protocol for MG-63 spheroid formation and culture is developed [60]. In brief, the device is first rendered to be non-adherent for the cells using surfactant of 1% (v/v) Synperonic^®^ F-108 (075711, Fluka, Sigma Aldrich, USA) in distilled water. The MG-63 cells are then seeded into the microfluidic devices using cell suspension with medium volume of 200 μl and density of 1.2·10^7^ cells/ml yielding a total cell number of 2.4·10^6^. Homogeneous and uniform distribution of cells in each culture chamber is achieved by seeding 200 μl of the cell suspension by pipetting to obtain about 480 cells in single chambers. In order to maintain the optimal cell culture environment, a medium refreshment step is performed every 12 hours. In the next step, the fresh medium with volume of 3 ml is introduced to replace the old medium and replenish the culture chambers. The device is incubated in a 37°C humidified incubator with 5% CO_2_. All the cell experiments are performed using fully grown and uniform-sized compact spheroids obtained on Day 2.

To visualize the structural development of the spheroids, fluorescence based live imaging is performed for the spheroids cultured in the microfluidic devices. Fluorescence nuclei stains Hoechst 33342 (62249, Thermo Scientific Inc., USA) and Cell Tracker^™^ Deep Red (C34565, Thermo Scientific Inc.) are used for staining nuclei and cytoplasm, respectively. In the experiments, the growth medium is replaced by a medium containing the stains with final concentrations of 5 μM 24 hours after the cell seeding. The staining solution is then washed out by the growth medium in the subsequent medium refresh step.

#### 2D monolayer cell culture

For comparison to the results obtained from the 3D cell spheroid culture model, conventional monolayer cell culture is also performed in the experiments. For the monolayer culture of the MG-63 cells, T-75 cell culture flasks (Nunc 156499, Thermo Scientific Inc.) are used. The cells are seeded in the flask with a population of 2.4·10^6^ and incubated for 48 hours with 6 ml of growth medium. The medium is refreshed every 12 hours, which is identical to the 3D cell spheroid culture. A confluent monolayer of the cells can be achieved after culture of 48 hours, and the cells are used for further experiments.

### Cellular stress application

In order to investigate the cell viability and cancer malignancy progression under different stresses, two types of essential cellular stresses that commonly observed in solid tumors are applied in the developed *in vitro* models in this research. In the experiments, the stress is applied from Day 2 to 4 during the growth phase of the cell spheroids on both monolayer and 3D spheroid cell culture models. In order to study the cellular responses under the stresses, cell viability is performed at the endpoint (Day 4) and the VEGF-A concentrations are quantified before (Day 2) and after (Day 4) the stress application.

#### Cellular stress 1—Nutrition deficiency

To study responses of the cells facing nutrient deficiency stress, growth medium with lower serum concentrations are prepared for the cell culture experiments. Compared to the 10% FBS (in volume) in the normal growth medium, the reduced serum growth media containing 5% and 1% FBS (in volume) are exploited to culture the cells in the experiments, while keeping all other constituents the same as the normal growth medium.

#### Cellular stress 2—Hypoxia induced factor (HIF) inhibition

In order to study effects of HIF inhibition on cell viability and VEGF secretion in the *in vitro* models, an anti-cancer drug, 3-(5’-Hydroxymethyl-2’-furyl)-1-benzyl indazole (YC-1), is employed to treat the cells in the experiments. YC-1 has been reported to suppress cancer cell VEGF secretion through inhibition of HIFs, and further slowdown the tumor proliferation [63]. In the experiments, YC-1 (0319S1-Y102, Sigma Aldrich, USA) is dissolved in dimethyl sulfoxide (DMSO) (67-68-5, Sigma Aldrich) to make 5 mg/ml stock solution stored at room temperature. The stock solution is diluted with the normal growth medium to the desired concentrations as working solutions within 24 hours.

Before the drug treatment on the cell models, titration of the drug is first performed on the monolayer MG-63 cell culture using a metabolic activity-based proliferation assay to estimate the half maximal inhibitory concentration (IC50). The detailed experimental procedure and results are described in the S1 File. In the experiments, the cell spheroids and monolayer cultured cells are treated with YC-1 from Day 2 with 3 different concentrations: 20, 40 and 60 μg/ml for 48 hours.

### Cell analysis

#### Spheroid size analysis

In order to study the spheroid growth within the microfluidic devices, spheroid formation and size distribution are monitored by microscope imaging every other day for 10 days. The cross-sectional diameters of the spheroids are estimated from the microscopic brightfield images for more than 20 individual spheroids within a single device. The average diameter is then calculated for the observation at each time point and normalized to the initial average spheroid diameter observed on Day 2.

#### Cell viability assay

To characterize viability of the cells under the different conditions, fluorescence-based cell viability assay is performed. Cell viabilities of the MG-63 cells cultured in the different formats under the stresses are evaluated using CellTracker^™^ Green CMFDA dye (C2925, Thermo Scientific Inc.) for cytoplasm staining or Calcein Violet^™^ (L34958, Thermo Scientific Inc.) as a marker for live and healthy cells with intact cell membranes, and Sytox Red^™^ (S34859, Thermo Scientific Inc.) is used to stain apoptotic cells having permeable cell membranes. In the staining process, the growth medium for both monolayer and 3D cell cultures are replaced with Live Cell Imaging Solution (LCS, A14291DJ, Thermo Scientific Inc.) containing live and dead stains with final concentrations of 10 μM and 5 μM for 30 mins, respectively. The cells are then washed three times with the LCS and observed using a confocal microscope (LSM 880, Carl Zeiss AG, Jena, Germany) and an inverted fluorescence microscope (DMI6000B, Leica Microsystems, Wetzlar, Germany). An imaging analysis software, Image J (Ver. 1.51j8, National Institutes of Health, USA), is used to quantify live and dead cells from images for the monolayer cultured cells and z-directional scanning of 3D spheroids. The cell viability is calculated as a ratio of the live cell number in the total cell population.

#### VEGF-A immunoassay

In the experiments, concentrations of VEGF-A secreted from the MG-63 cells in spheroid and monolayer cultures under various conditions are analyzed as indicators for tumor malignancy progression [64]. It has been reported that tumor cells secrete VEGF-A as an autocrine regulator promoting their proliferation and survival through tumorigenesis [65, 66]. In order to quantitatively investigate the VEGF-A secretion under different culture conditions, the conditioned medium is collected from the devices every other day during the medium refreshment steps. The medium is then centrifuged (1000 rpm for 10 minutes) and the supernatant is collected in test tubes and stored at -80°C for the following immunoassay. For quantitative measurement of the VEGF-A concentration, enzyme-linked immunosorbent assay (ELISA) is performed using a commercially available Human VEGF-A platinum ELISA kit (BMS277/2, Affymetrix, eBioscience) on the stored conditioned medium samples. In the measurement, absorbances at wavelength of 450 nm and 620 nm as reference are measured using a microplate ELISA reader (Synergy^™^ 2, BioTek Instruments Inc., USA). The VEGF-A concentration is quantified for the samples at different days, and the calculated values obtained from different days are normalized to that of the VEGF-A concentration before treatment (Day 2) under the same culture condition.

#### Statistical analysis

All measurements are performed independently for more than three times, and the results are statistically analyzed using a commercially available data graphing and analysis software, Origin^®^ 2018 (OriginLab Corporation, Northampton, MA). Statistical significance is evaluated by one-way ANOVA (SYSTAT, Systat Sofware, Inc., San Jose, CA) for all numerical data considering *p*< 0.05 as statistically significant.

## Results and discussion

### Formation and growth of cell spheroids

**Fig 3:**
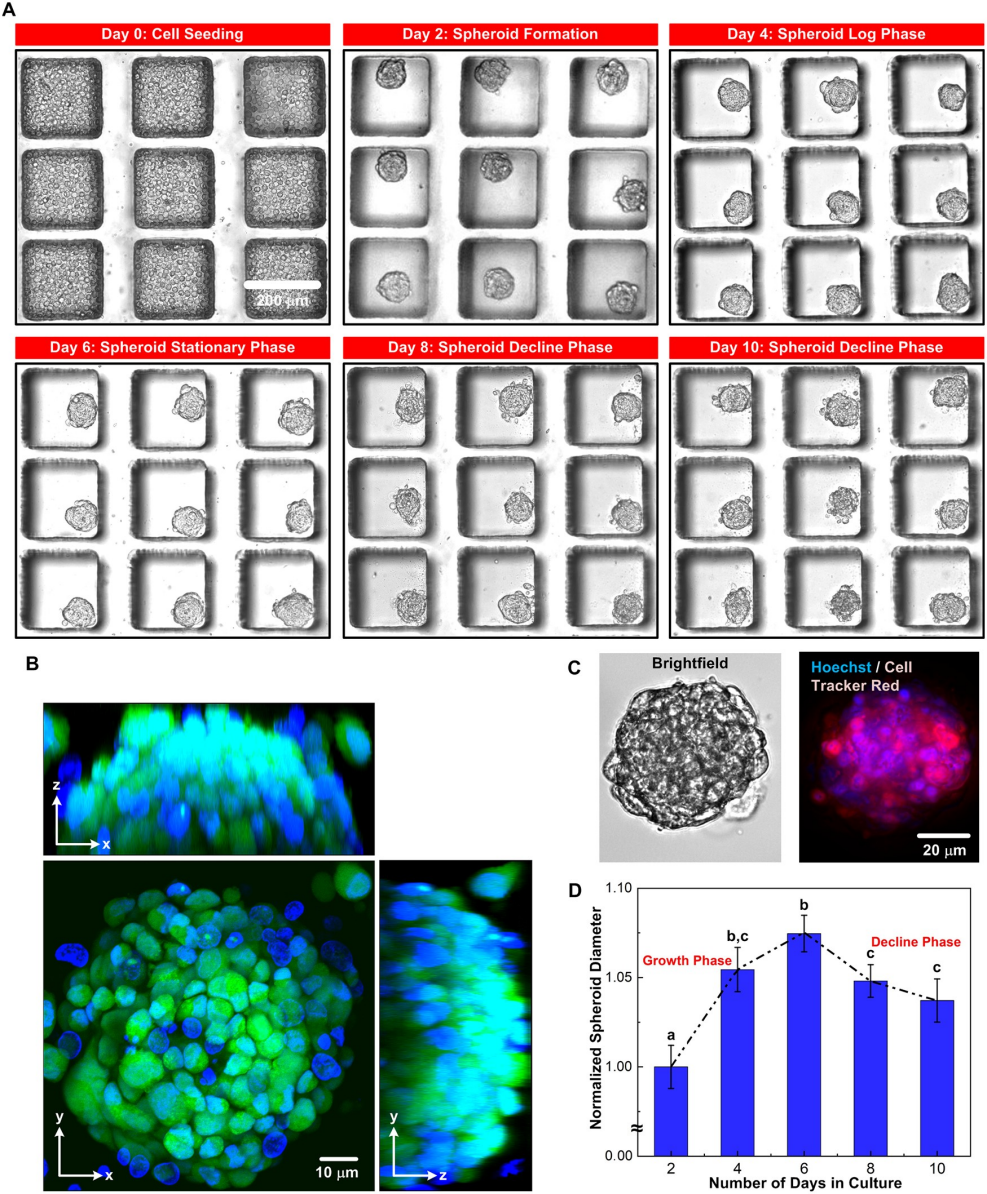
Growth kinetics of the spheroids in the microfluidic device. (A) Brightfield images of the MG-63 cell spheroids, cultured in the microfluidic device under 10-day observation period, including: cell seeding (Day 0), formation of the 3D spheroid structure (Day 2) and subsequent growth phase (Day 2 to Day 6), stationary phase (Day 6), and decline phase (Day 8 to Day 10). Scale bar is 200 μm. (B) Confocal images of a typical MG-63 spheroid stained with Hoechst for nuclei, Cell Tracker Green for cytoplasm, and Sytox Red for dead cells from different viewpoints. Scale bar is 10 μm. (C) Brightfield and fluorescence images of a typical MG-63 spheroid stained with Hoechst for nuclei and Cell Tracker Red for cytoplasm. Scale bar is 100 μm. (D) Plot of the diameters of the spheroids at different days, normalized to the spheroid diameter measured on Day 2. Data are presented as mean±s.e.m. One-way ANOVA is performed for statistical analysis, and the data with statistically significant difference are labelled with different letters (a, b, c = *p* <0.05).

Taking advantage of the designed microfluidic device, the MG-63 cells form dense and tightly packed uniform-sized spheroids on Day 2, with an average diameter of 85 μm, within the culture wells of the device, as shown in Fig 3A. The formed spheroids can be further cultured within the device for 10 days. Fig 3B shows the confocal images from different viewpoints of a spheroid harvested from the device after the 10-day culture. The images show that the cells can form solid tumor spheroids with less than 1% of cells showing red fluorescence from the Sytox Red suggesting the great viability of the cells after the culture [35, 67]. Further, the spheroid sizes are characterized by estimating the diameters from the collected brightfield and fluorescence images as shown in Fig 3C. Fig 3D shows the analyzed normalized diameters of the spheroids over the 10-day culture period in the microfluidic devices. In the analysis, diameters of the spheroids cultured in three different devices are measured from the brightfield images. The results show that distinct growth phases of the spheroids are observed over the culture period according to their morphology and size. The average diameter of the spheroids increases more than 5% from Day 2 to Day 4 indicating the rapid growth of the cells, which is designated as a growth phase. The average diameter slightly increases from Day 4 to Day 6 with the difference less than 2% suggesting the stationary phase of the spheroid growth. The average diameter decreases by approximately 3% between Day 6 to Day 10 and shows structural disintegration, which indicates the decline phase of the spheroid growth. The results show that our microfluidic device is capable of generating a large number of tumor spheroids with uniform size distribution where distinct growth patterns can be observed and quantitatively calculated at statistically significant numbers. Similar microfluidic spheroid cultures used to grow mesenchymal cell lines have shown to form significantly different spheroid sizes per culture chamber, with decline in growth of cells within the first 5 days [68]. Commercial devices (e.g. EZSPHERE and Nunclon Sphera) are capable of growing spheroids less than 1000 in number per device. The results show that our method is capable of culturing spheroids at large scale with precise size control where temporal phases of growth can be observed as a result of very high sample uniform sample size at same physiological conditions, and the samples can be further exploited for various biochemistry assays.

### Comparison between culture formats

**Fig 4:**
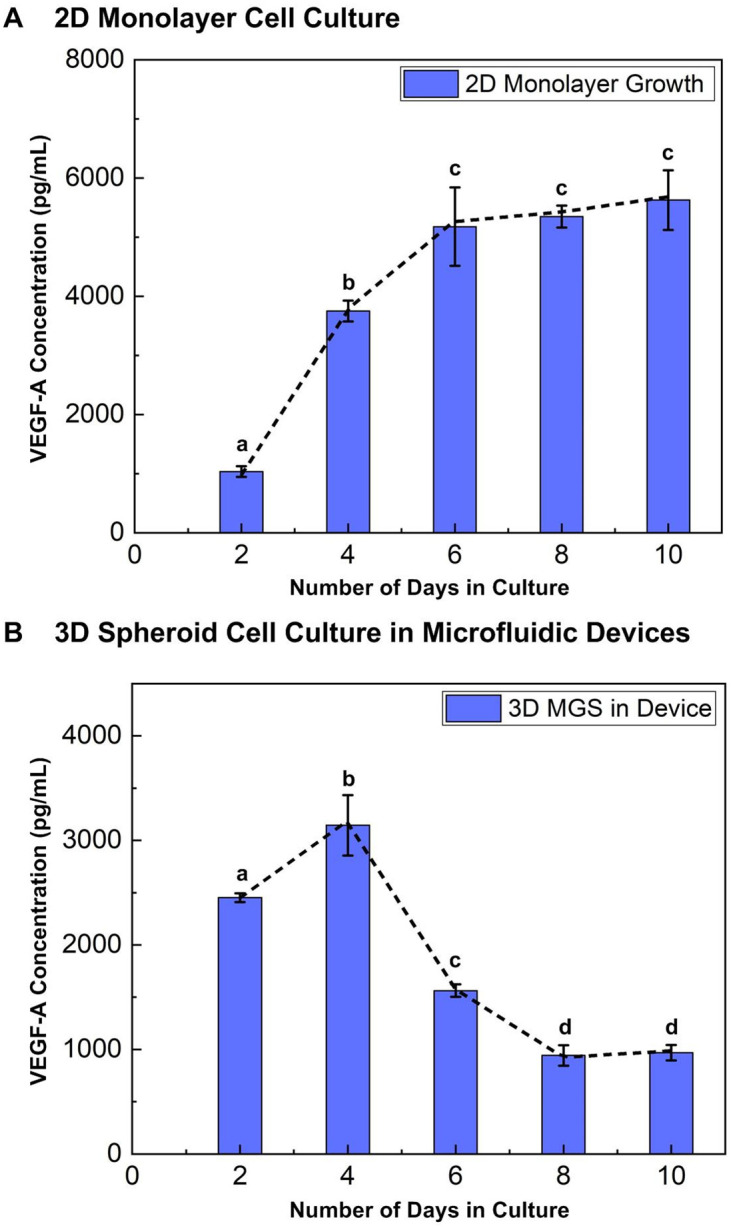
VEGF-A concentration measured from the conditioned medium collected from the monolayer and 3D spheroid cell cultures, and the calculated VEGF amount secreted from cells at different days of growth using ELISA. Data is presented as mean±s.e.m. One-way ANOVA is performed for statistical analysis, and the data with statistically significant difference are labelled with different letters (a, b, c, d = p<0.05).

To investigate the VEGF secretion profiles of the MG-63 cells in both 3D spheroid and monolayer culture formats, VEGF-A quantification using ELISA is first performed. Fig 4 shows the measured VEGF-A concentration of the conditioned medium collected from the monolayer and spheroid cell cultures at different days over the 10-day culture period, starting from 48 hours after cell seeding (Day 2). For the monolayer cell culture, VEGF-A concentration in the medium is estimated to be 1034.3±91.6 pg/ml on Day 2, increases steadily to 5176.8±661.6 pg/ml at Day 6 (Fig 4A) and remains similar from Day 6 to Day 10. In addition, the VEGF-A amount secreted from cells is also calculated and plotted for comparison. The VEGF-A amount secreted from the cells is estimated to be 206.9 pg/10^6^ cells on Day 2 and increased to 389.3 pg/10^6^ cells on Day 4, and slightly decreases and remains similar (287.2 pg/10^6^) from Day 6 to Day 10. The overall increase in VEGF-A concentration is a consequence of significant increase in cell population over the culture period [69–72]. In monolayer cell culture, the cells highly proliferate and secrete more VEGF at initial stage, and the cells reach confluence afterwards and secrete less VEGF-A during the culture.

In contrast, for the spheroid culture in the microfluidic devices, the plot shows that the VEGF-A concentration is 2451.9±42.5 pg/ml on Day 2, reaches maximum value of 3144.1±290.0 pg/ml on Day 4 and decreases rapidly to 968.5±73.5 pg/ml on Day 10 (Fig 4B). Similarly, the VEGF-A amount secreted from the cells is estimated to be 1038.9 pg/10^6^ cells on Day 2 and increased to 1135.1 pg/10^6^ cells on Day 4, and decreases to 368.2 pg/10^6^ on Day 10. The VEGF-A concentration and secretion patterns in 3D spheroid culture follows a similar trend to that of the average spheroid diameters shown in Fig 3B. The results show that growth of 3D spheroids under the closed microenvironment of the microfluidic device is affected by its size that is highly correlated with the cell numbers within the spheroid. Previous studies on solid tumors and spheroid models have also reported tumor size as an indicator of tumor progression and its relation with VEGF [66, 73, 74], which agree with the results from the experiments.

The difference in VEGF concentration and secretion profiles between monolayer and 3D cell cultures of equal cell seeding numbers, incubation period and culture conditions is attributed to their culture format and growth microenvironment [75, 76]. The VEGF secretion from the monolayer cells is less than that from the 3D spheroids in the initial 48 hours after cell seeding when cells are in growth phase. Studies comparing monolayer and 3D cell cultures have also shown VEGF expression of monolayer cultured cells to be less than that of the 3D cultures, which may be resulted from the more hypoxic microenvironments within the spheroids [52, 77, 78]. Day 2 to Day 4 is the growth period with maximum viability, proliferation and active growth in both monolayer and 3D microenvironments. Therefore, the time period is chosen to study the cell viability and VEGF secretion under different stresses.

### Effect of nutrient deficiency: Reduced serum culture

**Fig 5:**
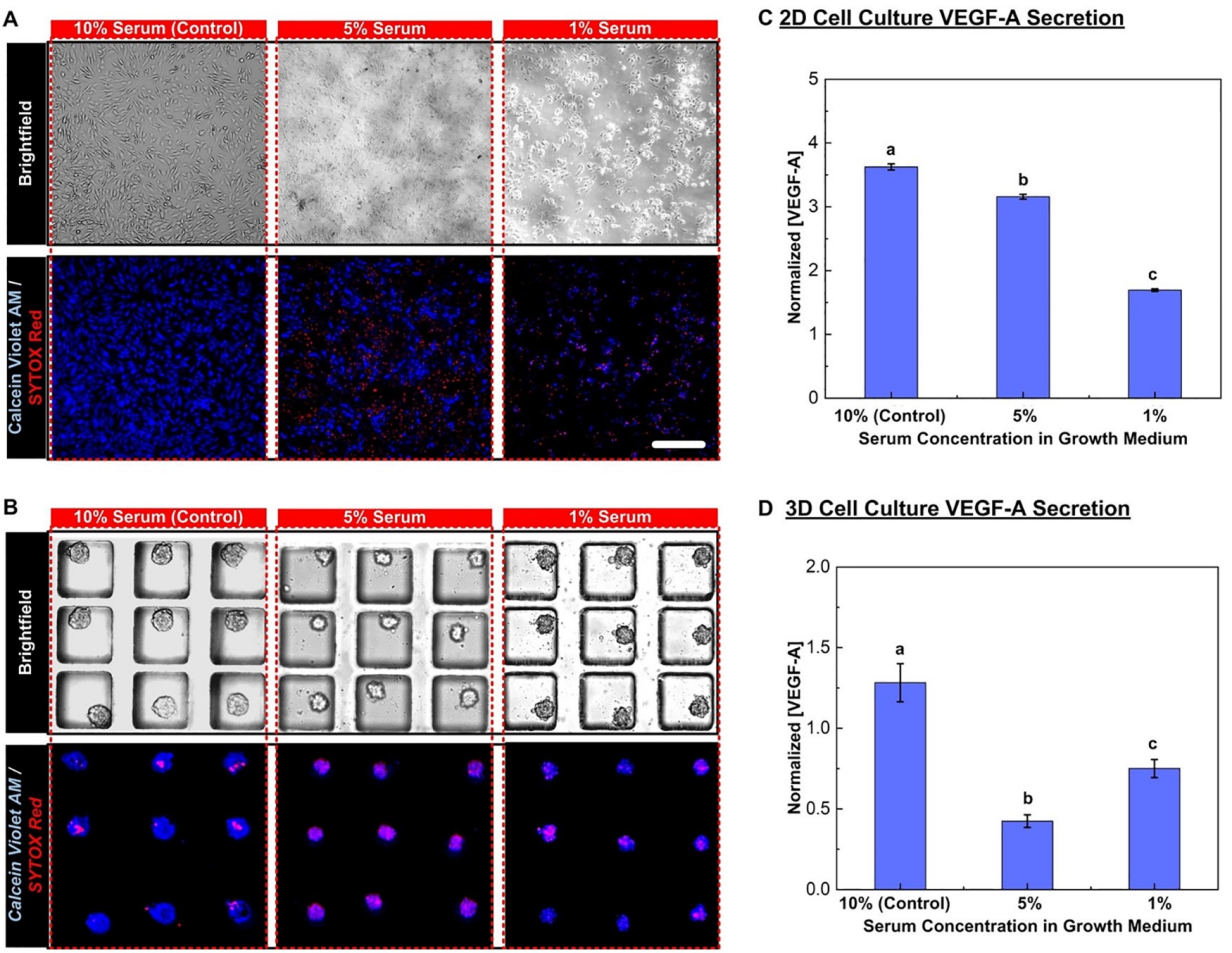
Growth of cell monolayers and 3D spheroids under different serum concentration in growth media. The treatment groups include samples cultured in growth media containing 5% and 1% FBS while samples grown in 10% FBS is control group. (A) and (B) show the brightfield and fluorescence images collected for estimation of cell viability for monolayer and 3D spheroid cell cultures, respectively. The cells are stained with Calcein Violet AM as a live cell marker and Sytox Red as an apoptotic cell marker. Scale bar is 200 μm. (C) and (D) show the VEGF-A concentrations and the amount of VEGF-A secreted from single cells measured at Day 4 normalized to those at Day 2 from the conditioned medium collected from the monolayer and 3D spheroid cell culture, respectively. Data are presented as mean±s.e.m. One-way ANOVA is performed for statistical analysis, and the data with statistically significant difference are labelled with different letters (a, b, c = *p* <0.05).

To investigate the VEGF-A secretion during growth of the MG-63 cells under the nutrient deficiency conditions, monolayer and 3D spheroid cell cultures are treated using the medium with the reduced serum concentrations of 5% and 1% in volume between Day 2 and Day 4 of the culture. Fig 5A and 5B show images of the monolayer and 3D spheroid MG-63 cells at Day 4 after the treatments, respectively. The cells are stained with Calcein Violet as a live cell marker and Sytox Red as an apoptotic cell marker in the images. In the monolayer culture, the total cell population and viability decrease when the serum concentration is reduced. Similarly, size and viability of the MG-63 cell spheroids also decrease under the nutrient deficiency conditions. The cell population and viability are further quantified by analyzing the fluorescence images, and the quantitative results are listed in Table 1. The results show that the cells proliferate much faster in the monolayer culture than those in the 3D spheroid culture. The total cell populations in monolayer cell culture, in groups at different serum concentrations are all greater than the initial seeding cell number, indicating cell proliferation.

**Table 1:** Total population and viability of the MG-63 cells, analyzed from the samples cultured with the reduced serum (nutrient deficiency) conditions. Data are shown as mean±s.e.m. (n = 3).

FBS Concentration (Volume)	Monolayer Cell Culture (Initial Population: 2.40·10^6^)		3D Spheroid Culture (Initial Population: 2.40·10^6^)	
	Total Population	Cell Viability	Total Population	Cell Viability
**10% (Control)**	13.81 ±0.07·10^6^	98.5 ±0.4%	26.27±1.18·10^5^	99.3±0.3%
**5%**	11.65 ±0.15·10^6^	51.5±1.7%	25.85±0.88·10^5^	66.7±3.6%
**1%**	9.02 ±0.29·10^6^	45.1 ±1.8%	12.90±0.23·10^5^	51.8±0.8%

Furthermore, the VEGF-A concentrations of the conditioned medium collected from the cell culture at Day 2 and Day 4, are quantified using the ELISA. In order to minimize the variation from the results obtained from different experiments, the measured results at Day 4 (after the treatments) are normalized to the concentrations measured at Day 2 (before the treatments) of the same experiments. In the analysis, two parameters: VEGF-A concentration in the medium and VEGF-A amount from single cells are estimated. The concentration can be exploited to evaluate the total response from entire culture cell layers or spheroids, and the amount secreted from single cells provides information of response from individual cells under the applied stresses. Integrating the analysis results, the possible underlying mechanisms of the cellular response can be further explored.

The quantification results obtained from the monolayer cell culture and 3D spheroid culture experiments under different serum conditions are shown in Fig 5C and 5D, respectively. For the monolayer cell culture, the results show that the normalized VEGF-A concentrations and VEGF-A amount secreted from single cells are all greater than one indicating the increased VEGF-A secretion that have resulted from the active cell proliferation after the 2 Day-culture under all the culture conditions. In addition, the normalized concentration in the medium monotonically decreases when the serum concentration in the medium is reduced, suggesting the nutrient deficiency stress negatively impacts the total VEGF-A secretion and may further suppress the tumor progression in the monolayer cell culture model. From the normalized amount of VEGF-A secreted from single cells, the value slightly increases when the serum concentration in the medium reduces from 10% to 5% (from 1.86 to 2.04) indicating the medium containing 5% serum promotes VEGF-A secretion from the cells although the proliferation may be slowed down leading to total VEGF-A concentration decreases comparing to the experiments conducted in the 10% serum medium.

In contrast, the normalized VEGF-A concentration in the medium is greater than one only for the control experiment in the 3D spheroid culture model. The concentration reduces more than 50% in the spheroids cultured in the medium with 5% serum, suggesting the suppression of VEGF-A secretion when the cells face nutrient deficiency, although the cell number can still increase. Interestingly, when the spheroids are cultured in medium with only 1% serum, the reduction in normalized VEGF-A concentration is less than that of groups cultured with 5% serum. In addition, the VEGF-A amount secreted from single cells remains closed to 1 (approximately 1.1) after the two-day culture in the 10% serum medium, and reduced for more than 60% (approximately 0.4) when the cells cultured in the medium with 5% serum. The results confirm that each cell secreted much less VEGF-A when facing the nutrient deficiency stress. In contrast, the normalized VEGF-A amount secreted from single cells increase to 1.4 when the serum in the medium is further reduced to 1% suggesting that the higher level of nutrient deficiency (1% serum) makes each cell secret more VEGF-A although it may lower the cell proliferation rate and viability. The results suggest that an intrinsic autocrine regulation of VEGF-A plays an essential role in the spheroid culture under extreme nutrient deficiency stress, which is not observed in the monolayer cell culture model. These results are similar to findings reported for VEGF-A and other cytokines in spheroids under serum deprivation [25, 35, 73, 79].

### Effect of HIF inhibition: Drug treatment

**Fig 6:**
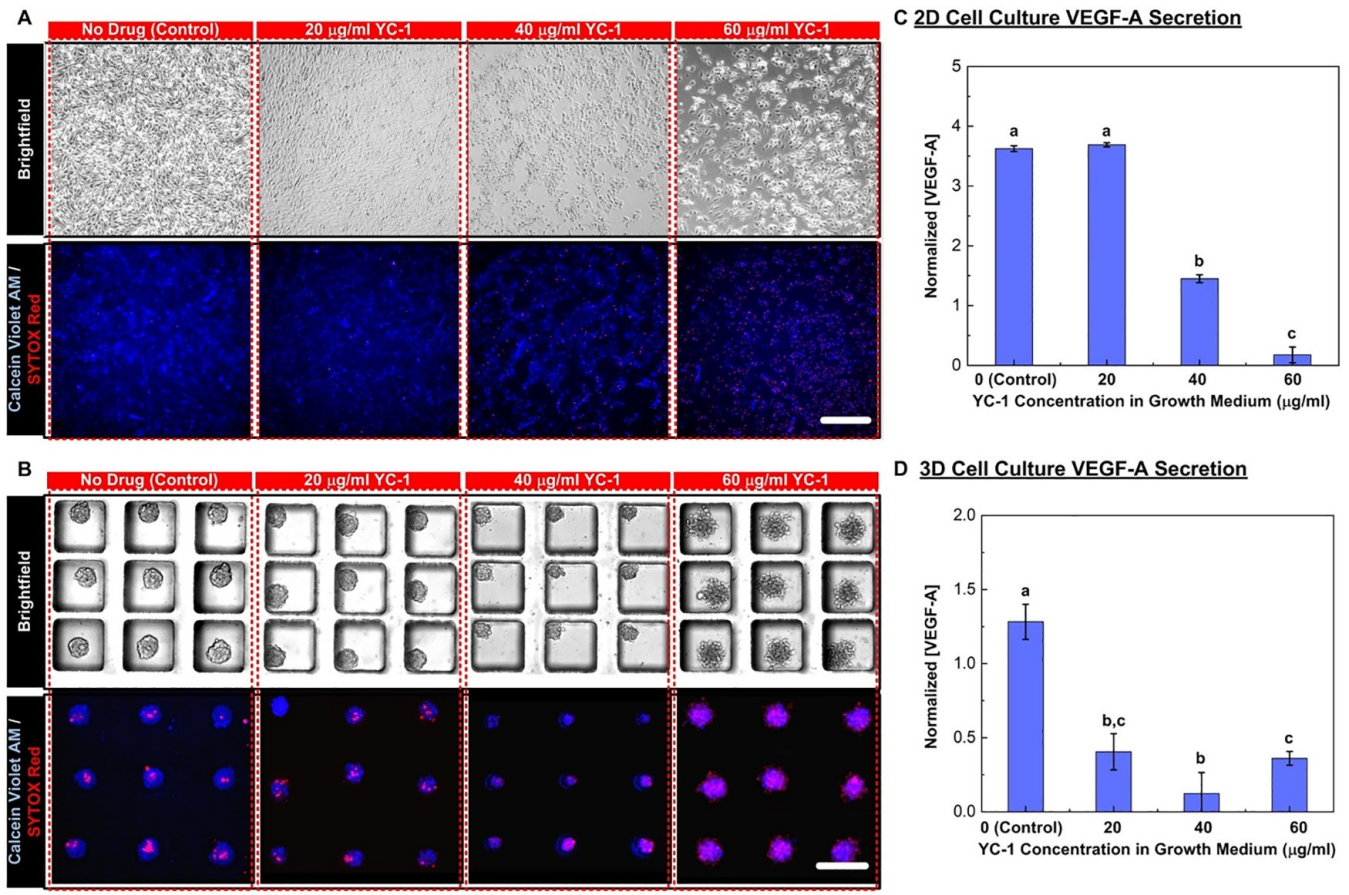
Growth of cell monolayers and 3D spheroids under different concentrations of HIF inhibitor YC-1 in growth media. The treatment groups include samples cultured in growth media containing 20, 40 and 60 mg/ml of YC-1 while samples grown without YC-1 is control group. (A) and (B) show the brightfield and fluorescence images collected for estimation of cell viability for monolayer and 3D spheroid cell cultures, respectively. The cells are stained with Calcein Violet AM as a live cell marker and Sytox Red as an apoptotic cell marker. Scale bar is 200 μm. (C) and (D) show the VEGF-A concentrations and the amount of VEGF-A secreted from single cells measured at Day 4 normalized to those at Day 2 from the conditioned medium collected from the monolayer and 3D spheroid cell culture, respectively. Data are presented as mean±s.e.m. One-way ANOVA is performed for statistical analysis, and the data with statistically significant difference are labelled with different letters (a, b, c = p <0.05).

To investigate the effects of the cellular stress with HIF inhibition, the MG-63 cells treated with the drug YC-1 with concentrations of 0, 20, 40, and 60 μg/ml in the medium from Day 2 to 4 in the experiments. Fig 6A and 6B show brightfield and fluorescence microscopic images of the cell viability reagents stained cells treated with the HIF inhibitor (YC-1) cultured in the monolayer and 3D spheroid formats, respectively.

In the monolayer culture, the total cell population and viability decrease when the YC-1 concentration is increased. Contrarily, size and cell population of the MG-63 cell spheroids does not decrease dramatically under the HIF inhibition conditions, but the morphology becomes different when the YC-1 concentration increases. The MG-63 spheroids begin to lose their integrity and the apoptotic cell number increases when the YC-1 concentration is higher than 40 μg/ml. The quantitative analysis results are summarized in Table 2. In monolayer cell culture, the total population and viability decrease monotonically when the concentration of YC-1 in the medium increases. In contrast, the cell viability of the cells within the spheroids is greatly reduced when cultured in the medium with higher concentration YC-1. Since the apoptotic cells are not detached from the spheroids, the total cell populations within the 3D spheroids cultured in medium with different concentrations of YC-1 remain similar. This suggests that the cells within the 3D spheroids are less sensitive to HIF inhibition than monolayer cultured cells, in terms of cell viability as observed in solid tumors and spheroid cultures [78, 80].

**Table 2:** Total population and viability of the MG-63 cells analyzed from the samples cultured with the HIF inhibitor, YC-1. Data are shown as mean±s.e.m. (n = 3).

YC-1 Concentration (μg/ml)	Monolayer Cell Culture (Initial Population: 2.40·10^6^)		3D Spheroid Culture (Initial Population: 2.40·10^6^)	
	Total Population	Cell Viability	Total Population	Cell Viability
**0 (Control)**	13.81 ±0.07·10^6^	98.5 ±0.4%	26.26±1.18·10^5^	99.3±0.3%
**20**	6.40 ±0.23·10^6^	92.7±2.6%	25.92±1.18·10^5^	88.7±0.8%
**40**	2.25±0.12·10^6^	79.0 ±2.2%	25.82±1.17·10^5^	78.4±2.6%
**60**	1.34 ±0.13·10^6^	40.8 ±1.9%	25.07±0.85·10^5^	51.9±4.5%

The VEGF-A concentrations of the conditioned medium collected from the cell culture are also quantified using the ELISA, and the normalized concentrations and the VEGF-A amount secreted from single cells are calculated by the experimental results after the experiments (Day 4) by those before the same experiments (Day 2). The quantification results obtained from the monolayer and 3D spheroid culture experiments under different YC-1 concentrations are shown in Fig 6C and 6D, respectively. For the monolayer cell culture, the normalized VEGF-A concentration and amount secreted from single cells are approximately 3.5 and 1.9 when the cells are cultured in the medium with no YC-1 (control), respectively. The cells treated with 20 μg/ml YC-1 do not show significant difference comparing to the control groups in VEGF-A concentration in the medium; however, the VEGF-A amount secreted from single cells increases to 2.9 indicating that the YC-1 promotes VEGF-A secretion from the cells although lower the cell proliferation rate and/or viability. Since the HIF expression from the conventional monolayer cell culture is little, the HIF inhibition drug, YC-1, may have limited effects on HIF inhibition of the cells. Furthermore, the promotion of the VEGF-A secretion by the drug needs further exploration to study the underlying molecular mechanisms. When the YC-1 concentration increases to 40 μg/ml, the normalized VEGF-A concentration decreases to approximately 1.5 (more than 55% decrease) and the VEGF-A amount secreted from single cells increases to 3.2 (more than 72% increase) compared to the control group, indicating that this YC-1 concentration begins to affect the total cellular response. Once the YC-1 concentration reaches 60 μg/ml, the normalized VEGF concentration decreases to almost 0 (decreased by more than 90% compared to the control experiments), and the normalized VEGF-A amount secreted from single cells also drops to 0.7. The results suggest that the YC-1 does help suppression of tumor proliferation and malignant progression at the high concentration in the monolayer cell culture model.

The results obtained from the 3D spheroid model show very different trends compared to those from the monolayer cell culture model. The VEGF-A concentration and amount secreted from single cells in the 3D spheroid model does not increase as much as that from the monolayer cell culture one in the control experiments. It has been shown that the cells proliferate slower in the spheroid culture which agrees well with the observation in the experiments. In addition, the cells are more sensitive to the HIF inhibition in the spheroid model due to higher hypoxia level within the spheroids. Under the lowest YC-1 concentration (20 μg/ml), the VEGF-A concentration and amount secreted from single cells reduce more than 60% after the 2-day culture, resulting in the normalized concentration and amount secreted from single cells of approximately 0.5 and 0.4, respectively. Furthermore, the normalized concentration and amount secreted from single cells reduces to less than 0.2 and 0.1 (decrease of approximately 90% with respect to the control group) after culturing in the medium with 40 μg/ml YC-1, respectively. However, despite the low cell viability, the experimental results obtained from the cells cultured in the medium with 60 μg/ml does not further decrease, and becomes similar to that from the cells cultured in the medium with 20 μg/ml. The results show that the VEGF-A concentration does not monotonically decrease when increasing the YC-1 concentration in the medium in the spheroid model which is distinct from those obtained from the monolayer cell culture experiments. VEGF-A suppression is observed to occur at a lower drug concentration in 3D spheroids than the monolayer culture.

In this study, the experimental results show contrasting cellular responses between monolayer and 3D spheroid cell culture models when cells are subjected to the cellular stresses. In the spheroid model, the apoptotic cell population increases under higher stresses. In contrast, the VEGF-A secretion from the cells decreases under the moderate stress and increases when the stress increases. The VEGF-A activity in the 3D spheroids suggests autocrine regulation leading to effective resistance against cellular stresses similar to that found in pro-angiogenetic tumors. Several studies suggest that HIF mediated autophagy is a cytoprotective survival strategy in tumors, which induces VEGF-A production to counter apoptotic cell death [45, 81]. The ability of converting cellular stresses into cytoprotective incentives induces metastasis in non-malignant tumors by promoting angiogenesis [13, 15, 81–83] where VEGF plays a significant role. From previous studies, the presence of HIF modulated low oxygen cores within osteosarcoma spheroids was reported [60]. In this paper, the developed osteosarcoma spheroid culture model illustrates VEGF-A activity similar in response pattern to that found during pro-survival angiogenetic activity of tumors, under both stresses: nutrient deficiency and HIF inhibition. The similar oxidative and chemical stressors have shown to upregulate VEGF activity and pro angiogenetic behavior in tumor spheroids in other works [84]. Therefore, the 3D spheroid culture developed in the developed microfluidic device mimics cytokine activity of tumors unlike the results to those obtained from the conventional monolayer cell culture model when systematically compared.

## Conclusion

In this paper, we develop a methodical approach to systematically compare cell viability and VEGF-A secretion under various cellular stresses of conventional monolayer cell culture and 3D spheroid culture models. The 3D cell spheroid culture is performed using microfluidic devices with great size controllability, easy operation which generates a perfusing and controlled fluidic space that is more analogous to conserved physiological microenvironments of solid tumors. The results are quantified using the imaging analysis and immunoassays and systematically compared between the two different cell culture models. In the experiments, two cellular stress conditions: nutrient deficiency and HIF inhibition are tested on the models. The experimental results show that the cellular responses including cell viability and VEGF regulation obtained from the 3D spheroids are different from those obtained from the monolayer culture. In the monolayer cell culture, cell viability and total VEGF-A secretion decrease when the cells face higher cellular stress. In contrast, the VEGF-A secretion from the cells cultured as 3D spheroids increases despite lower cell viability under higher stresses, which suggests that structural properties of spheroids are important parameters in regulating cytokines like VEGF under cellular stresses. Similar results have been also observed in an earlier work, where physical properties of 3D spheroids were studied as a controlling parameter of drug response [85]. The observations suggest that spheroids are capable of functionally sculpting their own microenvironments by secreting cytokines like VEGF in stressed situations of increased apoptotic death under high cellular stresses, which is different from 2D monolayer cultures. In this study, the results obtained from the 3D models are comparable to *in vivo* observation of tumor survival and can lead to fresh perspectives about tumor angiogenesis. For example, study of VEGF secretion using mice models have suggested a strong relationship between VEGF and tumor aggressiveness [65]. In addition, two independent studies compared VEGF secretion and non-metastatic osteosarcoma proliferation by clinical observation on patients, and suggested that poor prognosis and high local recurrences can be associated with boosted VEGF secretion [86, 87]. The approach developed in this study can also help to systematically analyze the autocrine activities of cytokines in tumors by replicating the conditions which lead to malignancy *in vivo*. Although the experiments conducted only using single cell type in this paper, the experimental results confirm potential applications of the approach to study cellular responses and underlying mechanisms based on the spheroid models for systematic comparison in various biomedical researches.

## Supporting information

S1 File (PDF)
