# Supplementary material for: Comparison of VEGF-A secretion from tumor cells under cellular stresses in conventional monolayer culture and microfluidic three-dimensional spheroid models
Source: PLoS One. 2020 Nov 11;15(11):e0240833. doi: 10.1371/journal.pone.0240833 (PMC7657494; doi:10.1371/journal.pone.0240833)
Supplement: S1 File — (PDF) [file pone.0240833.s001.pdf]

Supporting Information

Comparison of VEGF-A secretion from tumor cells under cellular stresses in conventional monolayer culture and microfluidic three-dimensional spheroid models

Sreerupa Sarkar,[1,2,3] Chien-Chung Peng,[2] and Yi-Chung Tung[2,3,4]*

Author Affiliations

[1]*Department of Engineering and System Science, National Tsing Hua University, Hsinchu 30013, Taiwan*

[2]*Research Center for Applied Sciences, Academia Sinica, Taipei 11529, Taiwan*

[3]*Taiwan International Graduate Program (TIGP), Nano Science and Technology Program, Academia Sinica, Taipei 11529, Taiwan*

[4]*College of Engineering, Chang Gung University, Taoyuan 33302, Taiwan*

Titration of HIF Inhibitor YC-1:

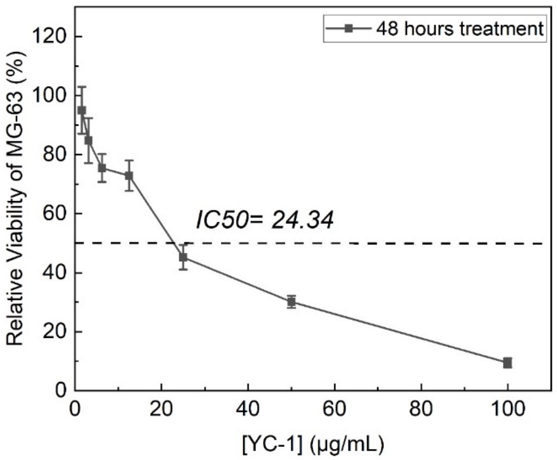

Figure S1: Plots showing relative viability of the MG-63 cells treated with different concentrations of YC-1 for 48 hours using 96-well plates. Cell viability is estimated using alamarBlue cell viability reagent[S1].

In order to estimate the half maximal inhibitory concentration (IC50) of the HIF inhibition drug YC-1 used in the experiments, the drug is first titrated against monolayer cultured MG-63 cells, and their relative viability is characterized using alamarBlue cell viability reagent (DAL1100, Invitrogen). The assay procedure is modified from the reported protocol[S](1). The alamarBlue reagent is used to detect metabolically active cells under different drug concentrations. Through the fluorescence-based measurement, the relative percentage of viable cells with respect to the control (no drug condition) can be estimated.

In the assay, 5000 MG-63 cells are seeded per well in 96 well culture plates with 100 µl growth medium. The cells are incubated for 10 hours under normal cell culture conditions. After cells attach and proliferate in the culture wells, the growth medium along with floating unadhered cells are aspirated out. Fresh growth medium with different concentrations of YC-1 are added to the wells with volumes of 200 µl and incubated from 48 hours. After the treatment, the growth medium is gently aspirated out and fresh growth medium mixed with 10% alamarBlue reagent is added. After 4 hours of incubation, fluorescence-based measurement is taken at 570 nm excitation, using a microplate reader (Synergy 2, BioTek Instruments, Inc., Winooski, VT). All the experiments are repeated five times for statistical analysis.

Figure S1 shows the relative cell viability (calculated with respect to the control groups) of MG-63 cells under different concentrations of YC-1. IC50 values is estimated as 24.3 µg/ml for 48 hour-treatment period. As a result, the drug with the concentration of 20 µg/ml is selected as the minimum concentration for the experiments using the cell monolayer and 3D spheroid models with the HIF inhibition stress condition.

References

S1. Eilenberger C, Kratz SRA, Rothbauer M, Ehmoser E-K, Ertl P, Küpcü S. Optimized alamarBlue assay protocol for drug dose-response determination of 3D tumor spheroids. MethodsX. 2018;5:781-7.
